# Supplementary material for: Accumulation of genetic variants associated with immunity in the selective breeding of broilers
Source: BMC Genet. 2020 Jan 17;21:5. doi: 10.1186/s12863-020-0807-z (PMC6969402; doi:10.1186/s12863-020-0807-z)
Supplement: Supplementary file 1 — Additional file 1: Table S1. Breeds and Naming Convention for the 15 Samples Analyzed in this Study. The main text uses the abbreviation to refer to each breed. Genomes sequenced from pooled blood samples are specified in brackets in the “Name” column. [file 12863_2020_807_MOESM1_ESM.docx]

**Supplemental Table 1. Breeds and Naming Convention for the 15 Samples Analyzed in this Study.**

The main text uses the abbreviation to refer to each breed. Genomes sequenced from pooled blood samples are specified in brackets in the “Name” column.

| **Study** | **Platform** | **Name** | **Abbreviation** |
| --- | --- | --- | --- |
| This study | Illumina NextSeq 500 | Ross 308 | Ross 308 |
|  |  | 1978 | 1978 |
|  |  | 1957 | 1957 |
| Fan *et al.*2013 (SRP022583) | Illumina Genome Analyzer II | Silkie | Silkie |
|  |  | Taiwan heritage L2 line | L2 |
| Rubin *et al*.*,*2010 (SRP001870) | SOLiD | Ross 308 (10 males) | CB1 |
|  |  | Undisclosed commercial broiler (10 females) | CB2 |
|  |  | Swedish layer (11 males) | WLA |
|  |  | Commercial White Leghorn (8 males) | WLB |
|  |  | High growth line (7 males, 4 females) | High |
|  |  | Low growth line (7 males, 4 females) | Low |
|  |  | Obese (10 males) | Obese |
|  |  | Rhode Island Red (8 males) | RIR |
|  |  | Swedish red jungle fowl (8 males) | RJFswe |
|  |  | Red jungle fowl | RJF |
